# “Even more than that, men love cars”: “Car guy” memes and hegemonic masculinity

**DOI:** 10.3389/fsoc.2022.1034669

**Published:** 2023-01-04

**Authors:** Lauren Dundes

**Affiliations:** Department of Sociology, McDaniel College, Westminster, MD, United States

**Keywords:** car guys, cars, masculinity, hegemony, memes, driving, gender

## Abstract

The construction of gender identities occurs through a variety of social forces, including memes widely circulated on social media. Beyond the function of internet memes as entertainment, they also promote gender-based bonding through humor in ways that encourage performative gender roles central to self-image. Decoding memes as a form of contemporary data reveals desires and fears, both conscious and unconscious, that underlie dramaturgical performances supporting hegemonic masculinity. In the case of “car guys,” car aficionados whose passion for cars is integral to their identity, memes reflect the group's aspirational presentation of self, including cars, as a symbolic physical embodiment of hegemonic masculinity. This semiotic study of 60 car guy memes shared on social media uncovered recurrent motifs centered around cars' ability to affirm men's position in the metaphorical driver's seat. Flashy cars were often portrayed as more desirable than women, a sentiment encapsulated by the meme, “Men love women, but even more than that, men love cars.” This novel analysis of memes explores the ostensible male preference for fantasy cars over emotionally risky relationships. Two salient themes relevant to conceptions of masculinity emerged: (1) car guys' apprehensions about male–female interdependence and (2) frustration with women's discretion in meeting men's emotional and sexual needs. Memes as a cross-sectional, unfiltered data source provide insight into the need to reconcile car culture with gender equality.

## Introduction

While cars possess symbolic and affective meanings that vary by demographic group (Best, 2006; Cross, 2018), car aficionados tend to “emphasize masculine powers and exclude women” (Walker, 1998; Walker et al., 2000, p. 153; Travers, 2008). These so-called “car guys” also known as gearheads, motorheads, and car fanatics, own (or aspire to own) muscle and sports cars. They take great pride in their cars and socialize with others sharing similar traditional values. Central to their identity is taking their putative rightful position in the “driver's seat,” a role perpetuated by “ambivalent sexism” and the stereotype that men are superior to women as drivers (Berger, 1986; Gaymard et al., 2022). Ambivalent sexism recognizes nuances of sexism by taking into account both beliefs that women are inferior (hostile sexism) and beliefs that they belong in traditional roles (benevolent sexism) (Glick and Fiske, 1997). This typology has relevance to a driver's gender in driving scenarios: Skinner et al. (2015) examined the relevance of ambivalent sexism to accident scenarios in which a defendant was depicted as navigating congested traffic. In this scenario, research participants who held hostile sexist attitudes assigned greater culpability to women compared to men. In another scenario that involved icy roads, participants high on benevolent sexism (that is, paternalism) were more likely to deem a woman responsible for the accident than a man facing the same conditions. This study exemplifies the potential empirical importance of understanding how sexism interconnects with conceptions of driving.

Group identity is also fundamental to understanding hegemonic masculinity in car guys. According to social identity theory, attitudes and behaviors are commonly based on group memberships (Tajfel and Turner, 1979). In the car guy subculture, hegemony reinforced by group identity provides a bulwark against identity threats, specifically challenges to masculinity. Even men outside of the car guy subculture tend to view interest in cars as a masculine credential. For example, Fisher (2009) found that when male nurses took care of male patients, that is, when they were being judged by other men, they enacted “culturally dominant masculinity... [talking] about blokey things [like] surfing and cars” including building hot rods (Fisher, 2009; p. 2672). This allowed them to assert their heterosexuality in a field in which they are in the minority. For car guys invested in car culture, however, passing references to cars are insufficient to bolster masculinity; instead, they emulate and identify with other car guys who saliently express their devotion to fast, high-performance cars (Reed et al., 2012). Their presentation of self is broadcast *via* their cars and driving styles as well as social media, providing outsiders with an opportunity to gain insights into their subculture of performative masculinity.

### Memes as data

This semiotic analysis involved decoding memes by interpreting salient expressive data of car guys. Memes as a window to social phenomena add to other forms of social media that provide new insights into the psychosocial aspects of groups (Kendall, 2002; Gal et al., 2016; Iloh, 2021). They are a participatory and creative reproduction of intertextual and remixed content, commonly disseminated on social media forums including Facebook, Twitter, Tumblr, YouTube, Reddit, and 4chan, among others, and a form of data that is “deeply entwined in the fabric of social life and discourse” (Iloh, 2021, p. 2). Memes provide insight into the values of those creating and consuming the qualitative content that is ripe for semiotic analysis [refer to Cannizzaro (2016) and Mahasneh and Bashayreh (2021) for contemporary applications of semiotics to memes].

### Study aims

This study leverages car culture's conspicuousness through memes in order to consider the following questions: (1) What attracts men to car culture? (2) How is a car conflated with a car guy's sense of self? and (3) How are these phenomena relevant to patriarchy? Despite limitations in analyzing car guy memes to suss out answers to these questions, this methodology provides an unobtrusive means to assess the subculture using data purposely shared in the public domain.

## Methods

Memes were selected from the yields of search engine queries using the keywords “Car guy memes” + images in November 2021 in the three most popular US search engines: Google, Bing, and Yahoo (Chris, 2021). These images, in turn, had been drawn from a variety of sources such as MemeGenerator (http://memegenerator.net), Quickmeme (www.quickmeme.com), and We Know Memes (http://weknowmemes.com/generator). Other sources yielded in search engine queries were https://awwmemes.com, https://www.memesmonkey.com, https://me.me/, https://ballmemes.com/, https://www.memecenter.com/, https://knowyourmeme.com/, https://carhumor.net/, and Pinterest. One of the most common sources of memes that appeared in search engine results is Car Throttle. Started in 2009 for young, millennial car enthusiasts, it claims to be the world's largest cross-platform automotive publisher, drawing from Facebook, Instagram, YouTube, and Snapchat with a monthly audience of 400 million people and a core demographic of males aged 18–34 (https://www.carthrottle.com/).

Memes expressing general subcultural values that revealed parameters of masculinity and those pertaining to car guys' view of women and/or dating were selected for analysis as long as the memes appeared in multiple but varied formats, a process that yielded 60 different memes, which are analyzed in this paper. For example, car memes dealing with brand loyalties were largely excluded as were memes related just to spending great sums on cars (if there was no mention of women or families). Similarly, many memes were out of scope as they were simply about how others do not share the same knowledge of or interest in cars, e.g., “Check engine”/Yup, it's still there [guy looks under the hood] or “How it feels when you talk to non-car guys about car specs” [guy in a restaurant booth staring ahead with a table pushed up against a blank wall].

Memes selected for the study were then analyzed for recurring salient themes, specifically themes that emerged from the data rather than pre-existing themes. The selected memes were then interpreted within the context of relevant literature. The following themes that emerged are examined in this paper: vehicles with a masculine persona, cars as a human body, car–human rear end equivalency, car–human rear end sound equivalency, performance as male power, women as symbolically equivalent to cars, memes in which a woman is valued less than a car, car guys in relationships with women, cars as a substitute for women, and car–women comparisons with cars as an implied sexual substitute.

The memes analyzed in this paper reflect dramaturgical masculinity, offering a contemporary means for “car guys” to succinctly “encode themselves... into the digitally mediated content they create, circulate, and transform” (Phillips and Milner, 2017; p. 195). In addition, using memes as an investigative tool can enhance our understanding of a subculture that has been largely neglected in the academic literature, with treatment that is “scant at best” (Martin, 2019; p. 81).

### Car culture described

Car culture revolves around a passion for cars, namely fast cars. Car guys tend to spend extensive time and money on their vehicles as a labor of love. Norms commonly include modifying or customizing cars, keeping them clean and free of scratches and dents, relishing and enhancing various features of their cars (including the engine power and noise), and admiring other standout cars. “Making their vehicle stand out from the rest” (Walker et al., 2000, p. 162) is a key aspiration of car guys. This description jibes with a video describing car guys by a car reviewer and self-professed car guy, Gold Pony (2015), whose YouTube channel videos boast over 60 million views (Gold Pony, 2021). Gold Pony calls reviewing cars an “addiction,” not just a hobby, consistent with a subculture known for an obsessive interest in cars (Zatz, 2019).

Car guys gravitate to muscle and sports cars, terms that are sometimes used interchangeably, despite differences between the two types of cars. Muscle cars, e.g., certain Camaros, Challengers, Chargers, Corvettes, Mustangs, and Gran Turismo Omologatos (GTOs) have an engine that is at least a V8, an eight-cylinder piston engine with more “muscle” than cars with four or six cylinders for fuel intake.^1^ These American-made two-door sports coupes have powerful engines designed for high-performance driving and are associated with patriotism (Lezotte, 2013). Sports cars, such as particular models of Porsche, Ferrari, Lotus, or Lamborghini cars, tend to have recognizable sleek body styles, with elements reminiscent of race cars. They are smaller and lighter than muscle cars, winning them notice for acceleration, speed, and performance (e.g., excellent handling to maneuver curves) (Wes, 2021).

Admiration of these vehicles builds camaraderie among car guys but also conveys that insider status depends on authenticity, namely values, attitudes, and behavior that are “real” or genuine, as a common meme reflects: “Real car guys respect other car guys” although several variations exist that add an insider–outsider proviso by adding the words, “Except Honda. Nobody likes them”.^2^

### Car culture in societal context

There are several reasons why car symbolism has greater resonance for men than women. Historically, a woman's place was as a homemaker and a housewife, with driving presumed to pose a temptation to shirk domestic responsibilities (Morgan, 2009). Discouraging women from considering cars to be part of their domain served to inculcate traditional gender roles and encourage their dependence on men (Franz, 2011). In turn, men's governance of car matters became conflated with their sense of autonomy and control that stymied women's physical and metaphorical mobility. The expression “where the rubber meets the road” affirms how driving connotes power, which for male drivers, cements their status as paterfamilias. It would be remiss to omit the obvious aspects of car culture that are associated with masculinity such as the “exhilaration of driving at high speed, the great pleasure in out-accelerating a rival vehicle when the traffic lights turn to green, of overtaking “every vehicle on the road,” and of having a beautiful girl sitting alongside in the front passenger seat” (Walker et al., 2000, p. 164). The term “passenger princess” refers to a car guy having an attractive woman in the passenger seat as a type of ornamental accouterment, with her function understood to be to a foil to the driver's dominance.

Gendered aspects of car culture are also notable in motor contests that resemble updated chariot races (Kottler, 2010), events where women drivers threaten the macho image of the sport (Matthews and Pike, 2016). This applies to various male-dominated professional races, such as National Association for Stock Car Auto Racing (NASCAR), with stock cars that are ordinary cars modified for racing, as well as Formula 1, race cars with a single seat.^3^

Women like Janet Guthrie, who in 1976 became the first woman to take part in a NASCAR Winston Cup Superspeedway Race, had trouble finding sponsorship due to exclusion from the old boys' network. About 25 years later, (now-retired) professional racer Danica Patrick gained more traction but was also acclaimed for her pin-up posters and the 2008 *Sports Illustrated* bikini shots in which she “unapologetically used sex appeal to promote herself” (Ross et al., 2009; para 43). Embracing stereotypical femininity included breast implants, “during her second full-time year in the NASCAR Cup Series, at age 32 to “have the whole package,”” (Henderson, 2022; para 4). Thus, Patrick's inability to project hypermasculinity led to her deference to the sport's embodiment of traditional gender roles in which part of “the package” for a woman entails catering to the male gaze.

### How vehicles inculcate a masculine persona

Other manifestations of cars' ability to confer masculinity appear in the lucrative *Fast and Furious* film franchise as well as the video game *Grand Theft Auto* (GTA), in which masculinity is defined by both sexual prowess and displays of aggression (Gabbiadini et al., 2016). In the case of the former, the word “fast” is slang for sexual promiscuity while the word “furious” can mean “violent or intense.” Similarly, GTA relies on female characters that tend to be sexualized and subordinate to male protagonists, reinforcing traditional gender stereotypes (Gestos et al., 2018). In addition, Hollywood reinforces the nexus between cars and male power by linking being without a car and abstinence in males, e.g., in *The 40-Year-Old Virgin* (2005) in which actor Steve Carell plays the title character who commutes on his bicycle (Hiskes, 2010).

In the popular Disney-Pixar *Cars* franchise, with anthropomorphized talking cars, the star is a Corvette-inspired car, Lightning McQueen. McQueen is a nod to Steve McQueen, star of the 1968 film *Bullitt*, who drove a muscle car, a Ford Mustang GT, renowned for its use in a famous car chase scene on the streets of San Francisco.^4^ Furthermore, car chases and other types of competitive driving are *de rigueur* in many action-adventure movies (and featured in the popular *Fast and Furious* film series).

This theme also pertains to the iconic, fictional, and animated Speed Racer of the mid-1960s, whose Mach 5's front end is patently phallic, with its prominent rocket-shaped central protuberance. Although Speed Racer's car exists in the fantasy realm of animation, vehicles' sexually suggestive imagery may at times be less symbolic and more overt in terms of their representation of a man's body: “Truck nuts are fake testicles that hang down from the back bumper of a truck, usually from the hitch... In the mid-to-late 2000s, the product hit a tipping point and truck nuts exploded [in popularity]” (Lamoureux, 2015, para 12).

One of the most blatantly sexual parts of muscle and sports cars is the manual stick shift, “a metaphor for a man's phallus” (Morgan, 2009, p. 53). For example, movie car chase scenes usually include various camera angles that show a man with his hand on the gear shift, indicating the driver's finesse in controlling his stick, without relying on automatic gear shifting that takes control away from the driver.^5^ Interestingly, the word “drive” in noun form is an innate, biologically-determined urge to attain a goal or satisfy a need.

**Figure 1:**
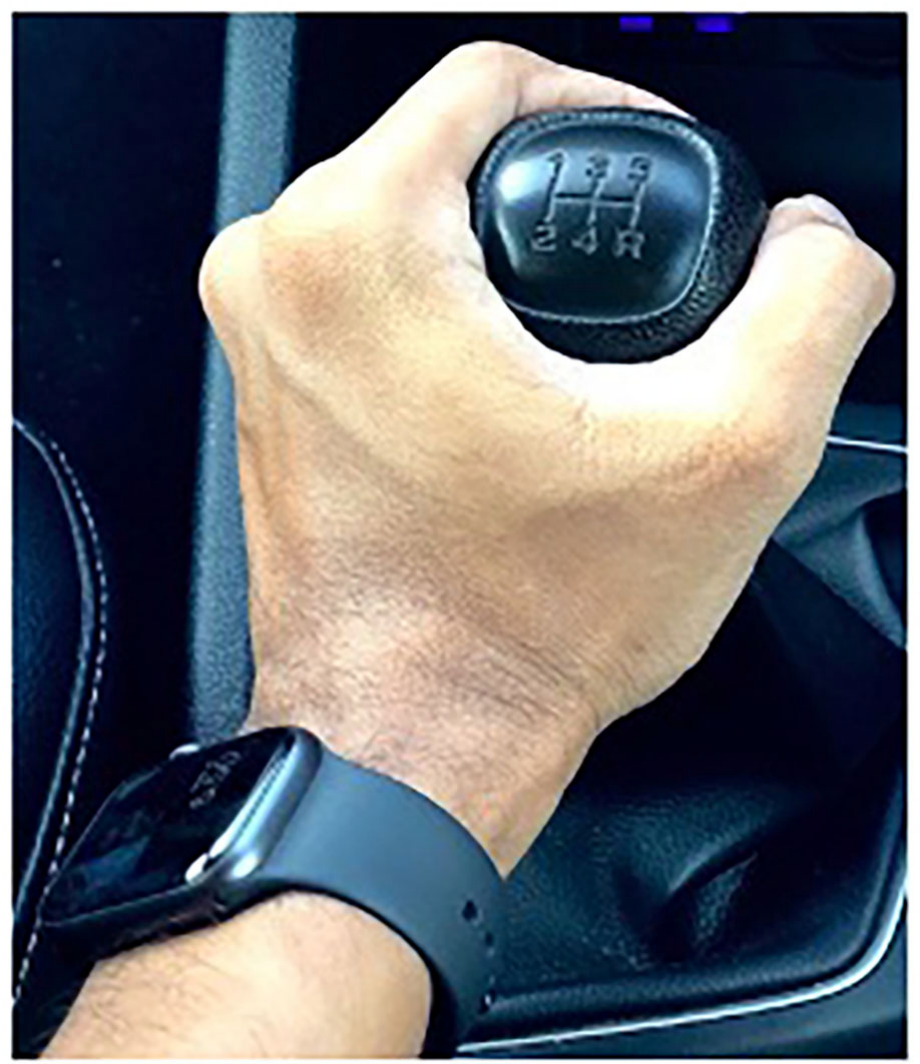
Real cars. Photo credits for meme: photo by Dicson @smartdicson, https://unsplash.com/license; https://unsplash.com/photos/5o9h0mRLI-0.

The following meme expresses that automatic cars are not “real” cars, presumably because they have lost their masculinity without the power conferred by the stick shift. Refer to Figure 1: Real cars do not shift themselves. This meme is a riff on the well-known book by humorist Bruce Feirstein, *Real Men Don't Eat Quiche* (1982), which satirizes stereotypes of masculinity. Cars without stick shifts could be seen as symbolically castrated since once such a car is in “drive,” no manipulation of the gear lever is needed, resulting in a loss of phallic control and a blow to masculinity.

Another meme treats the stick shift not only as increasingly rare but as a living being.

Endangered species

[picture of a gear shift grid]

A manual transmission requires the use of a clutch, a pedal used to connect and disconnect a vehicle's engine from its transmission, a word associated with stereotypical masculinity. Being a “clutch player” means to perform well at an important, high-pressure moment, based on the idea that a clutch facilitates control over a situation (Clutch, 2018).

Furthermore, muscle cars are arguably factory-made hot rods (specially modified vehicles designed for extra power and speed). A rod is “a time-honored phallic symbol” (Rashkow, 2000; p. 84; Lee and Johnston, 2015) while the term “hot” is associated with sexual readiness and attractiveness. This meaning was blatantly apparent in the 1971 “Dickmobile” hot rod on the cover of rock band Steppenwolf's “Ladies Only” album. Similarly, revving an engine to warm it up incorporates revving, with a metaphorical meaning to excite a person (Rev, 2022), complete with sound effects. Burnouts (a maneuver in which wheels spin in place with friction creating visible smoke) are arguably a form of “visual special effects” denoting heat that signifies how a driver is literally and metaphorically “smoking,” slang for sexually attractive. These associations begin early, as with the brand “Hot” Wheels, toy cars marketed to boys (Hourigan, 2021).

More overt symbolism exists related to modifying a hot rod to boost its torque using a stroker kit (with the word stroke tied to male masturbation and other slang sexual meanings). Suck, squeeze, bang, blow is a double entendre referencing the internal combustion engine cycle completed in four piston strokes (a suction stroke, a compression stroke, an expansion stroke, and an exhaust stroke), with symbolic meaning captured in a meme:

Suck, squeeze, bang, and blow

[Sudden Clarity Clarence template with guy looking confused]

And it's not a porno movie

### Cars as a human body

While cars can connote masculinity due to one part or in their totality, there is also support for cars as extensions of humans by virtue of various car parts having equivalents in the human body, as the term “muscle car” suggests. Cars as “cathected, humanized machines” (McLeod, 2020, p. 234) undergo repairs at “body” shops.^6^ In this sense, the polysemic nature of cars means that they may not only connote phallic power but also may represent a human body, including a surrogate woman. In other words, the gender-related symbolism of the car varies according to context (as with the polysemic flames emoji that signifies either anger or sexual passion).

“Head” lights imply that the front of the car is a head, while head “lights” function as eyes (as the car's headlights make the road visible just as the iris and pupil control how much light enters the human eye). The front-end car grill for ventilation functions like a mouth (air exchange) just as a grill on a person can signify teeth ornamentation. In fact, one blogger wryly notes that “There's an entire corner of the auto accessory industry built around the realization that the front of a car looks like a face. People who never quite get beyond that realization seem to do mainly two things with that information: Buy tickets to every movie in Pixar's “Cars” franchise and put “carlashes” on their vehicle” (Notte, 2021).^7^ Similarly, a *Fiat Canada* ad makes use of how car headlights and grills resemble faces (refer to the ad titled “Drive Friendly” posted on Imgur, a social media site for hosting and sharing images: https://imgur.com/0riLfcL). This phenomenon also appears in memes:

[Conspiracy Keanu template]

If a dude missing an eye is driving a car,

Is he considered driving on one-headlight?

[photo of Vin Diesel, lead actor in *Fast and Furious* series]

She's gotta have those kind of eyes

[front end of white Mazda Miata]

The hood, called a bonnet (in the UK), also connotes that the front end of a car is a head. A hood is a head and neck covering while in Scotland, a bonnet can mean a cap for men or boys while a (feather) bonnet is a type of military headdress worn in Scottish Highland infantry regiments. Similarly, consistent with a car as an ersatz human body, tires, the means for locomotion, would be symbolic feet, consistent with a “flat tire,” slang for when a person steps on the back of another person's shoes. Big Foot tires are specialized, oversized tires sold to the military and civilians alike (with a possible unconscious selling point related to the (unproven) belief that shoe size correlates with penile length). This could also explain why a wheel clamp or wheel lock is commonly called a boot, with cars violating parking rules being “booted”.^8^

### Car-human rear end equivalency

The rear end of the car has “tail” lights and a “tail” pipe for exhaust gas. Tail is slang for buttocks and one meaning of a trunk is posterior, as in the expression “junk in the trunk,” slang for fat in the buttocks (typically referring to a woman) (Idioms, 2021).

Various memes treat a car's rear end as belonging to a human, e.g., in the case of tailgating:

Oh, you want to ride my ass?

[template of Gene Wilder as “Condescending [Willy] Wonka”]

Please tell me how that will make the car in front of me drive faster

### Car-human rear end sound equivalency

The desire to make the conspicuous “rumble” of a car's exhaust even louder unconsciously replicates sounds of human bodily functions, specifically with the tail pipe emitting a sound comparable to passing gas. In animals, the tail is located near the rear end, making the fumes from the tail pipe positionally analogous to mimicked human flatulence, an act and sound that is more acceptable among males (Haslam, 2012). Flatulence has also commonly been compared to the power of thunder, from Aristophanes' *Clouds* and Chaucer's *Miller's Tale* to a movie about a boy with uncontrollable flatulence called *Thunderpants* (2002); it may be related to the naming of the Thunderbird muscle car as well.^9^

Passing gas also has latent meaning as a form of anal power, as with bullroarers, an instrument used in a range of different cultures, exclusively by men, to produce the sound of flatulence (Dundes, 1976).^10^ Thus, the volume of ersatz flatulence discharged by men in their cars brings welcome attention to car guys, but it is not a sound that women produce or mimic to gain stature.

The role of exhaust in attracting notice is the subject of memes.

I do not always just sit and listen to my exhaust

But when I do, so does the whole neighborhood

[rear end of muscle car]

The rumble is reminiscent of race cars and commonly marketed to men in ads that cater to the “male fantasy of being a race car driver, the ultimate symbol of combustion masculinity” (Redshaw, 2018; p. 91).

Allowing drivers to make their cars louder is a marketing gimmick that plays on conceptions of masculinity: “With the push of a button, the soundtrack is mechanically enhanced to give the car more of a V8 exhaust growl [while] the throaty rumble enhances in sport mode” (Williams, 2019). This concept aligns with a description by an online car-buying service: “Enhancing the sound of your exhaust gives a different, but equally satisfying, sense that the vehicle you are driving is a force to be reckoned with” (CarsDirect, 2012).

While loud cars may have broad appeal to men, they are especially appealing to car guys. Various memes celebrate their pride in making sure they are heard, as well as their willingness to flout noise regulations and even pay fines for the privilege of announcing their presence. In fact, disdaining the authority of their archrival, the police (Walker et al., 2000), is a value-added bonus to gaining notice, as the meme below conveys.

I know it is loud. Write the ticket

[template of actor Leonardo DiCaprio throwing money]

Similarly, another car noise, honking a horn, can bolster masculinity. Hitting or blasting the horn conveys aggression, like the horn of an animal that poses a threat. Not coincidentally, the word horny has a sexual meaning, showing that horns conflate sex and aggression.

### Performance as male power

Combustion masculinity that entails auditory signaling is closely linked to car “performance” and sexual prowess featured in car ads (Wilson, 2020). This assertion can be confirmed by a simple web search using the words “male performance” that will yield pages of websites related to erectile dysfunction. The emphasis on 0–60 in X seconds can be decoded as the ability of a man to be in a state of readiness in mere seconds, banishing fears of a metaphorical, and public, display of symbolic impotence.

The designation 0–60 is called “pick up,” referring to both how fast a car accelerates and starting a conversation with someone with the goal of having sex. Although this measurement of acceleration is a major selling point, certain 0–60 claims actually require a specially-prepared drag surface or are simply misrepresented.

Car marketing capitalizes on these male performance fears and desires, suggesting how driving certain cars embodies masculine ideals, separating men from boys and men from women in a way that is purposefully exclusive, as Avery (2012) describes:

His Porsche becomes a magical transport, bringing him back into the game and enabling him to capture the attention of women... [since].... only “real men” can handle the car (p. 327).

### Women as symbolically equivalent to cars

While cars provide a means to exhibit the qualities of a “real man,” whether women can fulfill the same need is more complicated: they may either enhance virility or undermine male dominance. Men in romantic relationships must engage in “give and take,” a reality of relationships that *ipso facto* gives women power. Thus, a man's reliance on a woman for fulfillment makes him dependent on her, a quality at odds with hegemonic masculinity. However, if cars can substitute for women as needed, then men have a backup plan that protects them in the event that a woman undermines their authority, as suggested by the meme, “Men love women, but even more than that, men love cars.”

Better yet, as a pre-emptive strike, car guys may unconsciously promulgate the notion of cars as superior to women on social media and elsewhere, treating sports and muscle cars as an acceptable fallback if a woman threatens their dominance.^11^ Support for this interpretation lies in “sports car as girlfriend” memes. In the example below, the equivalency is based on the financial drain imposed by both a girlfriend and a sports car:

With girlfriend                        Without girlfriend

[Picture of an empty wallet]   [Picture of an empty wallet]

+                                              +

[Picture of nondescript            [Picture of sports car]

sedan]

A similar meme has the same equivalency theme, but escalates the sexism:

Having a turbo car is like having an

Obsessive girlfriend

[overly attached girlfriend template]

High maintenance costs

And always needs attention

In the above meme, women are again compared to cars, but this time referencing their allegedly excessive emotional needs. Furthermore, the meme also implies that women who need a lot of attention are “obsessive” (that is, neurotic). Women's desire for attention may contrast unfavorably with their traditional role as nurturers who meet but do not make demands.

In another car–woman equivalency meme, a man defines a partner's love as passively accepting her status as (only possibly) equal to her partner's prized possession:

I just want a woman who's nice enough that I actually

Couldn't choose between her and my car, but who loves

Me enough to never ask me to

Another meme points out how men and women think differently when it comes to cars:

Her: He's probably thinking about other girls

[picture of a couple in bed: a woman looks over at the man with his back to her as he stares ahead pensively]

Him: Are mechanics just doctors to cars?

The above meme compares women who are inclined to spend time thinking about such matters as relationships, fidelity, and attractiveness to car guys who are preoccupied with their car and its welfare rather than their significant other. Another popular meme has a similar message:

Girls are like “All guys think about is sex”

[red sports car]

Bitch please

Similarly, in the video of Rihanna's *Shut Up and Drive* (winner of the People's Choice R & B category in 2008), Rihanna is virtually ignored by the two men who are concerned only with winning the imminent drag race. Despite her chorus chant to “shut up and drive” combined with suggestive gyrations, her invitation for sexual intimacy is ignored while the two men featured are solely concerned with winning the race, with their hands shown on their stick shifts. In other words, the word “drive” means sex to a woman while for men the word can entail competition with other men, prioritized as a means to establish the pecking order in a hierarchy of masculinity.

### Memes in which a woman is valued less than a car

The next memes acknowledge women as desirable, but still as -less- desirable than a flashy car:

If you had the choice of your dream girl or your dream car

[red sports car]

What rims would you put on it?

Women may also be treated as an expendable commodity that is inferior to a car:

Why no girlfriend?

[sports car]

Because he has a bitchin' race car!!!

This meme also includes thinly veiled misogyny, suggesting that a bitchin' (cool) car is better than a bitchin' (complaining) woman, an interpretation based on how the word bitchin' is slang for either remarkably bad -or- good, depending on context (Bitchin', ND).

In the subsequent meme, women's #7 ranking shows their placement as patently subordinate to cars, exacerbated by the expectation that a woman should love cars, in accordance with a car guy's interests.

10 things I want in life:

1. Cars.

2. More cars.

3. Car friends who like cars.

4. A big garage for all my cars.

5. Money for my cars.

6. Cars.

7. A woman who loves cars.

8. A big trailer for my cars.

9. A track for my cars.

10. Cars.

Other memes overtly communicate how the value of a woman compares to that of a car:

Roses are red, violets are blue,

[sports car]

I love my car, more than you

Similarly-themed memes provide context for this preference, namely the perils of relationships:

The more I learn about relationships...

[sports car]

The more I love cars

While this meme does not specifically articulate that cars are preferable to women, it hints at this eventual conclusion based on problems with relationships. When considered in conjunction with the next meme, it seems likely that fraught power dynamics common in relationships explain the preference for cars, specifically playing on the stereotype of women as mysterious, unpredictable, and hard to manage.

I love cars more than women

When a car has a problem, the mechanic sorts it out

But when a woman has a problem, you wouldn't know until it's too late

The next meme conveys a preference for cars, but instead of blaming relationship difficulties, it implies that driving the car satisfies a man's sexual needs.

I love my car more than my girlfriend

[picture of a screw blower]

It has a big blower

Blower terminology is loaded with sexual innuendo. A screw blower is a supercharger that brings maximal air into the motor to increase combustion output using a screw compression element, comprised of male and female rotors to boost engine power (Humphreys, 2013).

Superchargers (like turbochargers) feed into inflating a driver's stature: “Fast cars and even pretty cars are all about horsepower. One of the best ways to get there is with a supercharger. There's no better way to draw a crowd around your car than with a blower sticking through the hood” (Smith, 1998; para 1). These modified cars, sometimes with a big block engine mounted in the middle, connote a sense that the engine (or its metaphorical phallic counterpart) is too big and powerful to contain.

When car guys do reference their sex life, a lack of emotional commitment to women emerges as a theme. The following meme that references mechanical expertise likens sex to working on cars while at the same time conveys disrespect for sexual partners:

I screw

I nut

I bolt

It's tough being a mechanic

[Two kinds of screws and a nut]

Screw is a slang verb for having sex while nut as a verb (as in the meme above) is slang for ejaculation. Anatomically, bolts have a head, and a nut is screwed onto the threads at the bottom, which could explain the expression “nuts and bolts” meaning “essential,” that is, if the two items are the symbolic equivalents of a penis and testicles, that would be prized in patriarchy. The line, “It's tough being a mechanic” is sarcasm given how the meme's “hit it and quit it” description of casual sex is blatant sexism that glorifies men's avoidance of commitment and commodifies women as a means of sexual gratification.^12^

### Car guys in relationships with women

The previous memes apply to men who are not in committed relationships. However, car culture memes also reflect concerns of car guys with partners. The memes below reiterate the wish that women could understand car guys' devotion to cars and presumably their preference for spending money on activities such as car customization rather than on a wife or family.

They said the GTR was not a family car...

[guy loading groceries into the back of red sports car]

So I got rid of my family

Interestingly, the next meme acknowledges women's power, as conveyed by the word “let” (i.e., allows):

Behind every great driver

Is an even greater wife who lets him buy car parts

However, the man in the above meme is identified as a “great driver” (active) while the wife's “greatness” is not only passive but also defined as deferring to her husband's wishes, consistent with the definition of hegemonic masculinity in which women become inured to “accommodating the interests and desires of men” (Connell, 1987; p. 183). This pseudo-praise of an “even greater wife” is a form of ambivalent sexism (Glick and Fiske, 1997) with its ostensibly positive tone (referring to a wife who is “even greater” than her husband) that in actuality requires a wife to accede power in spending-related decisions (to defer to her husband's hobby).

The following meme also relates to a couple's spending, but involves circumventing spousal input:

Quick! Gotta make it look dirty

[guy working on a car engine]

Before the wife notices it's new!

The term “the wife” (above) depersonalizes a man's spouse and implies her conformity to a traditional role. It also suggests that wives: (1) should be deceived, (2) can be deceived by only a simple ploy, and that (3) they are an obstacle to men enjoying a favorite pastime.

Memes can also convey car guys' love of cars, chosen over amorous activity:

[Woman]: Babe, it's raining out. Let's rent a movie and cuddle

[Woman lies on a bed in lingerie, holding a remote control]

Never got the movie

[Sports car driving through the flooded parking lot, creating a big spray of water in its wake]

The following five memes reflect how car guys' love of cars can escalate into a desire for multiple cars that demarcates men from women:

I have too many cars

Said no guy ever

Just one more car

I promise

Bought a new car

And the wife asks

[Ray Liotta laughing in *Goodfellas* template of two men laughing uproariously]

If I'm going to sell the old one

And then she said

[Ray Liotta laughing in *Goodfellas* template of two men laughing uproariously]

Why do you need more than one car??

OCD

Obsessive car disorder

An ethnography of car culture revealed that a man with a “car obsession” sometimes justified the resources he expended on excessive cars by “discovering” an appropriate vehicle for his wife's use (Lezotte, 2013, p. 92), co-opting her into his hobby that was at times obsessional. Expenses for some may even include “car condos,” car storage units marketed to men “who sometimes struggle to persuade spouses of the wisdom of plunking down $300,000 for a garage” (Green and Zahler, 2014, para 5), a type of “lavish retirement home for their cherished vehicles” (Green and Zahler, 2014, para 9).

Even women that condone or support cars guys' predilections may be reminded of their status relative to their husband's preferred car:

If he lets you drive his car

[woman sitting behind the wheel of a car]

You better feel damn special

Notably, the above meme starts with the word “if,” implying that a woman may or may not be permitted to drive a spouse's beloved vehicle. In fact, even “‘girl racers” feel compelled to tolerate “compliance with the subordination of women... to accommodate the interests and desires of men” (Lumsden, 2010, section 2.7).

In an extreme display of woman vs. car, women that cannot understand this car fixation are expendable:

When she says that she doesn't like the car

[picture of sports car]

The sad time has come where

You must part with the girl

The woman is also called a “girl” above, a way to demean a woman who does not support her partner's avid interest in cars.

### Cars as a substitute for women

Car guys' love of cars may also be escalated to the point where a car is portrayed as a surrogate romantic interest:

The rings she wants [2 overlapping wedding bands]

The rings I want [four round illuminated taillights]

Related memes liken a man's feelings about his car to “popping the question” (getting engaged):

If you like it, then you need to put a ring on it

[Picture of a car's o-ring used for gaps in a gland's mating hardware]

Note: a gland is a sleeve used to seal a piston rod or other shaft.

[Man bent on one knee, facing a red sports car, extending a wheel rim toward the car]

... She said Yes!

Proposing, whether to a woman or a car, implies possession and ownership that enhance virility (Dundes et al., 2018). Once a car guy ties the knot, his car may still be a priority, as the meme below indicates by the groom using the treasured (and expensive) wedding gown train to clean his tires (possibly symbolic feet):

Car guy priorities

[Bride stands next to a car as groom bends down to use her wedding gown train to polish his wheel rims]

Various other memes have a “no explanation necessary” approach to a car substituting for a woman:

They said I'd be alone on Valentine's Day...

[A guy leans on the roof of the red sports car, with arm outstretched on top of the car]

[A dozen roses have been placed on the hood of the car]

They were wrong

Other memes state the car-woman hierarchy of importance unambiguously:

There's nothing more    Except this GTR. It puts out

powerful than love        2,000 WHP^*^

[couple embraces on     [muscle car]

the beach with palms

at sunset]

                                       ^*^WHP = wheel horsepower

In other words, cars, especially fast cars, confer sufficient masculinity that they may be preferred over a relationship with a woman:

□ Single
□ Taken
■ In the garage building a fast car and do not have time for your drama

In another version of the above, the last box reads instead: “In love with my car.”

Not only do cars also apparently offer the advantage of escaping women's alleged mood swings and histrionics, but they also allow men to avoid women altogether and still project masculinity, as the next meme implies:

Guys that drive these

[An array of muscle cars]

Never have a girl in the passenger seat

Other memes explain why women are non-essential, if not dispensable: their power to damage the ego of their male prospects or partners.

I would rather be alone with my car...

Than be with someone that can't accept me for who I am

Always remember

Your car will never wake up one day and tell you it doesn't love you anymore

We may spend too much money on car parts

[muscle car]

But at least our cars won't leave us and say “I don't love you anymore”

In contrast, the problems cars pose do not jeopardize the man's self-esteem:

When you love your girl,

But she has

[sports car at the gas pump, with the gas nozzle in the tank]

Drinking problems

**Figure 2:**
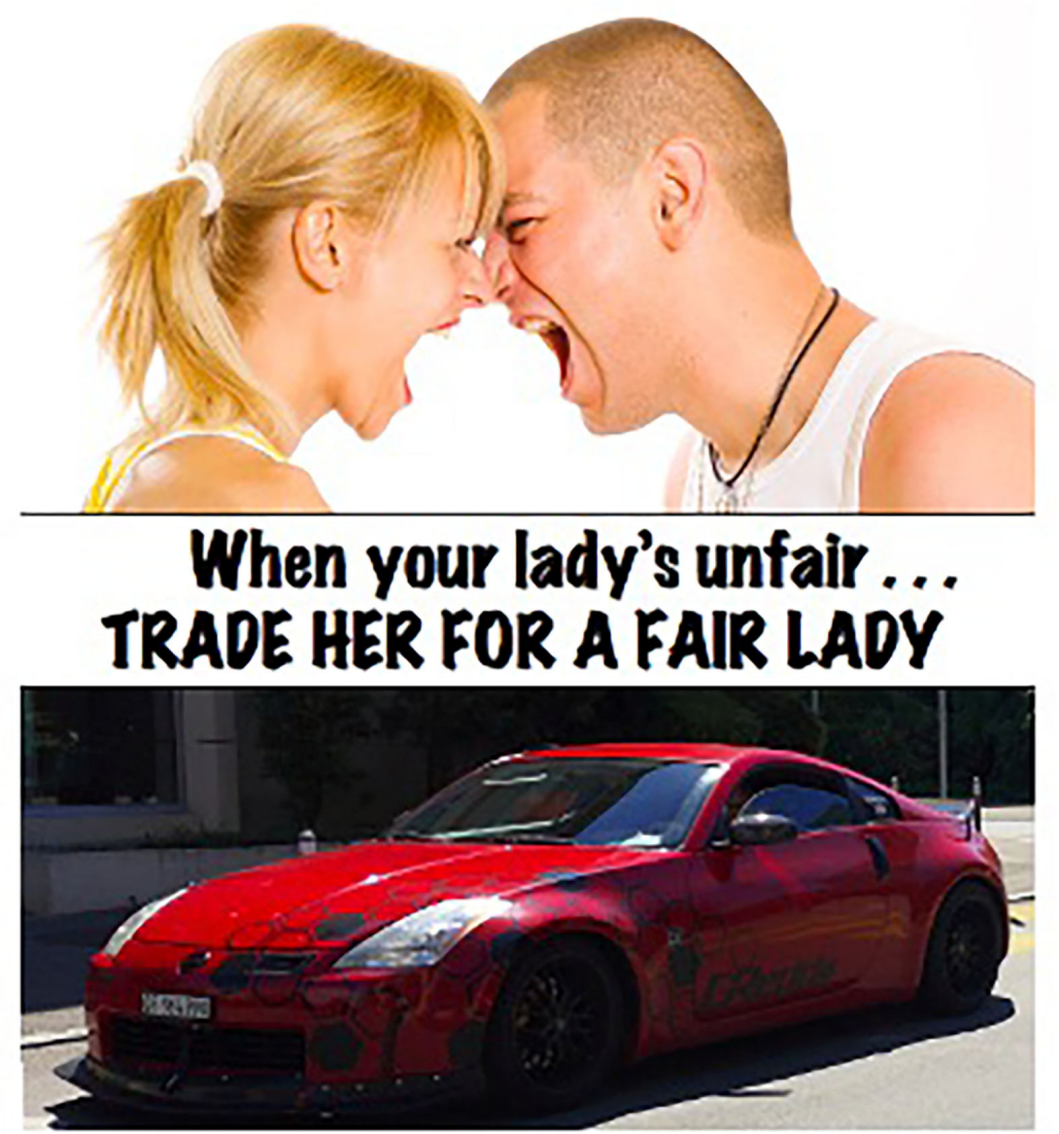
Trade her for a fair lady. Photo credits for meme: Vic on Flickr: couple arguing: https://creativecommons.org/licenses/by/2.0/; https://www.flickr.com/photos/59632563@N04/6238711264; Crash71100 on Flickr: Nissan 350Z: https://creativecommons.org/publicdomain/zero/1.0/; https://www.flickr.com/photos/152930510@N02/51247645444/in/photolist-2m5zzxS-UWj2Jb-2n8xZE7-2neWCj6-4QP6pt-MVwYLu-RsN3Y6-2gvNLqF-2nfTboQ-6B2ae9-2naKuY9-6fLtQp-2mVy5qs-2gwqBSm-2jsAaqs-QKm51R-2jcDhWF-Y5bGX5-2jbR1aL-2gK89MA-2mVuiZv-2iH8fSx-2iPb5kP-2iH3QVD-49Gvek-MQ9WR9-2iH6Ekn-2iH8niD-2jbPEVV-2n1xK7Y-2iPb5mA-2j6auEf-2jJVfAZ-7ibKJs-26Wpr4U-cX7t1Y-2iH3QJ1-Ma1v7h-2iH3RbU-4LyYig-2iH3QCj-2mXqBUV-2iH8hDi-2iH6AMa-2iH8fb2-2iH8cg4-2iH3Yss-2iH6AGf-2iH6ADQ-2iH3UDL.

When women are not so manageable, cars apparently present a viable option as a replacement, as in the meme above that refers to a fairlady (the name of the Nissan 350 Z, a two-door sports car, manufactured from 2002 to 2009).

Refer to Figure 2: Trade her for a fair lady.

### Car–women comparisons with cars as an implied sexual substitute

While significant others in car culture require special handling, memes can also express a “hostile” form of sexism, essentially misogyny, in which women are compared to cars as commodified sex objects (that still cannot necessarily compete with the allure of fast cars).

Here we see one of the most beautiful things on earth

[Woman in a leotard and high heels leaning suggestively into the window of a sports car

Her face, seemingly irrelevant, is not visible]

The other wears high heels

I like my women the way I

Like my track

[Racecar driver on a racecourse]

Wet and Curvy

Women are like cars

Every time you get one...

A newer and better model comes on the market...

[sports car]

Women are like cars

You pick one with the lowest mileage and the best looking

[sports car]

Women are like cars

They must belong to one owner, otherwise, it's public transport

[sports car]

Not driving your Corvette to keep miles low is like

Not banging your girlfriend to keep it tight for the next guy

[Corvette]

The next meme provides context for the previous memes, explaining why car guys portray women as expendable commodities, useful for sexual gratification:

Only gives you pleasure when she wants

[young woman]

Gives you pleasure when the boost kicks in

[sports car]

The meme above clearly decries women's ability to withhold sex, a right relevant to the #MeToo and Yes Means Yes (affirmative consent) movements. In contrast, cars offer no resistance or threat to male dominance; they only enhance it. This leads to car substitutes as a way to avoid rejection, maintain control, and enhance virility.

While cars can substitute for women in projecting masculinity, women are still necessary for their role in procreation. However, there is some meme evidence that fantasy “conceptions” of cars include wishful thinking in how this reproductive role might become obsolete.

[Mercedes trunk opens and the front of another miniaturized Mercedes emerges from the trunk]

Version 1 caption: Cervix dilated, head is engaged

                               Ruptured membranes, crowning

Version 2 caption: How cars are made

                               Nature is beautiful

This meme that circumvents women's role in birthing arguably guides the interpretation of the next three memes that do not directly reference women, but imply that cars, and not relationships, are essential for happiness, making women optional:

The real key to happiness

[Car key dangling in front of a sports car]

Happiness isn't around the corner

It -is- the corner

[Muscle car going around a sharp bend]

Whoever said “money can't buy happiness”

Bought the wrong car

**Figure 3:**
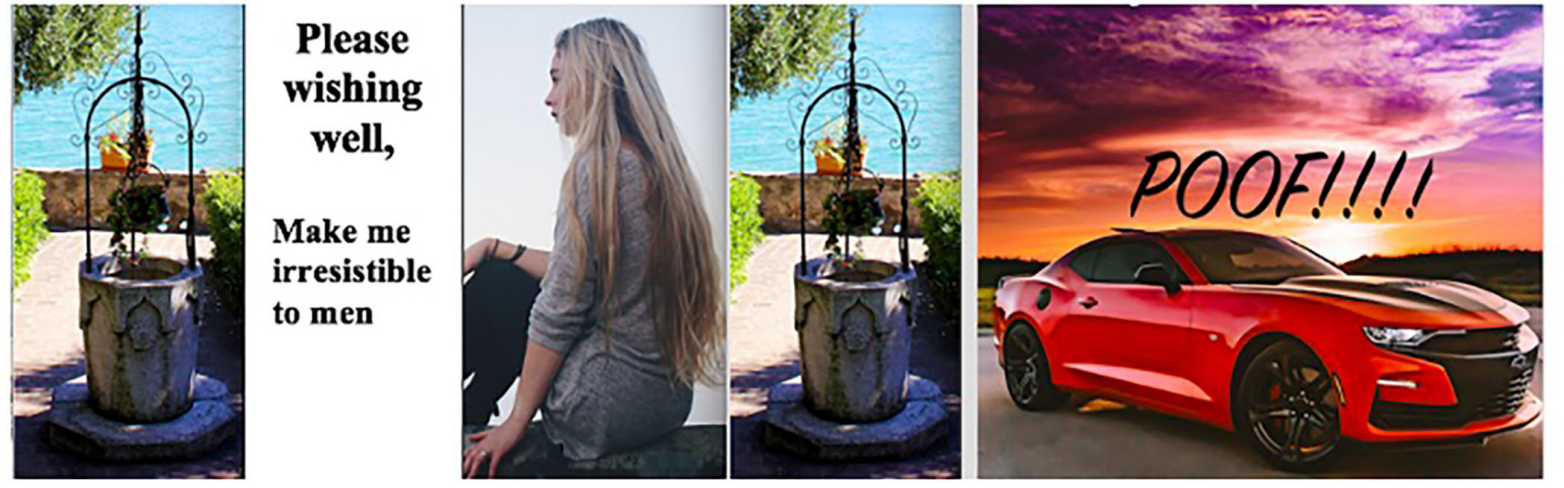
Please wishing well… Photo credits for meme: Francesca Minto on unsplash: wishing well: https://unsplash.com/es/fotos/-H38UIFvodo; https://unsplash.com/license. Zachary Staines on unsplash: woman sitting: https://unsplash.com/photos/JMKkm3leBKE; https://unsplash.com/license. Yuvaraj Singh on unsplash: car https://unsplash.com/photos/EqPF4QT60j4; https://unsplash.com/license.

Taking this point a step further, if it is cars and not women that make men happy, and women are difficult, demanding, and fickle individuals who also abuse their power as sexual gatekeepers, then the right kind of car is more desirable. See Figure 3: Please wishing well...

This meme arguably pits the woman against the car, as a competitor of sorts. In addition, the woman is shown as (1) naïve to what men really want and (2) incapable of becoming more alluring than a fast car even with the help of magical powers. The words “impossible to resist” are consistent with an inexorable car obsession in which needs and wants along with aggression and libido have free reign. The car transmogrifies into a mistress of sorts, which car guys may modify to be “pimped out” or “tricked out,” perhaps unconscious references to prostitutes who offer sexual gratification without commitment or judgment. This could also relate to the perception that when men marry, they are “settling down,” giving up the “high octane” excitement of going into “overdrive” and living life “in the fast lane” to accede to expectations of “the wife” or mundane responsibilities associated with a committed relationship in which they are “spinning their wheels”.^13^

## Discussion

Despite the advantages of memes as an unfiltered data source, a major limitation is an inability to identify the creators and the consumers of the material. Fortunately, however, prior studies of car culture can provide insight into factors relevant to how the car guy subculture is intertwined with hegemonic masculinity. Walker et al. (2000) found an inverse correlation between boys' interest in cars and school among the working class, suggesting that weak academic performance prompted the youth to seek “power and authority... denied them in most other realms” (Walker et al., 2000, p. 159). Similarly, Hatton (2007) found that car culture was alluring to unemployed or underemployed young men who sought to boost their masculinity in response to feeling otherwise devalued. In addition, there is some evidence that “boy racers” of higher social classes are considered effeminate by their working-class counterparts (Lumsden, 2010). Thus, car culture may confer manhood to compensate for feelings of “exclusion from the labor market and exclusion from the academic curriculum” (Walker et al., 2000, p. 162):

“[M]otor vehicles provide a cultural medium in which young men, whether or not they are physically small, labeled “dumb” by others, or are from a vilified ethnic or racial group, can demonstrate masculine strength, virility, and prowess: their technical ability to control a ‘performance’ motor vehicle at high speed and their courage and daring through risk-taking” (Walker et al., 2000, p. 162).

Missing from Walker et al.'s insightful analysis quoted above, however, is the aspect of the corporeality of cars as a surrogate human body. In this regard, research on athletes is relevant. When amateur rugby players' bodies fell short according to a masculinity yardstick, the resulting threat to their identity as team members resulted in embodied remedial identity work, in which various means of using their bodies served to reclaim hegemonic masculinity (Giazitzoglu, 2022). Likewise, in car reviews, masculinity may be equated with a car in its physical form. “Rumble Seat” columnist Dan Neil described his experience test driving a “fantasy automobile,” the McLaren 765LT (longtail)^14^ retailing for $429,000 and boasting 755 horsepower (far exceeding the typical 180–200 horsepower car). Neil touts its “muscularity... tendons and sinews” commenting, “I felt like handing it a robe,” implying that its body reminded him of a muscular naked man that “creates a vortex of attention” (para 2, 15) (Neil, 2021). Similarly, with car guys, the car serves as an extension of the body that both projects masculinity and provides an alternative means to attain status, especially in the absence of other accessible avenues to achieve recognition.

## Notes on methods

Memes offer an opportunity to gain insight into the members of a subculture, a method that avoids social desirability bias in which research participants may feign support for politically correct views (Munsch and Gruys, 2018) or alter their behavior when under observation (Hawthorne effect). Furthermore, standard data collection methods cannot avoid major limitations in attempts to tap into inner thoughts (including unconscious beliefs).

It must be emphasized that the 60 memes analyzed in this paper are only a snapshot of data available in November 2021 and are not intended to suggest a monolithic hegemonic archetype of the “car guy” subculture. The variations and progress in addressing hegemonic masculinity emphasize the importance of documenting masculinity in a variety of contexts, longitudinally (Nichols, 2019). Furthermore, the ever-changing cultural context relevant to car culture will inevitably lead to new memes that reflect the changing zeitgeist.

Despite the limitations of the methodology employed, these memes show how flashy cars may serve as an outlet for men to express repressed fantasies of power, freedom, visibility, respect, and sometimes domination. Thus, car culture permits escapism in the form of socially sanctioned passion for cars in which men conflate their identity with that of a powerful car. The value of a muscle car projects strength that exemplifies corporeal masculinity as a cultural performance, conveying autonomy that minimizes or disparages any power that women have over men. By identifying with their cars and idolizing traits associated with ostentatious vehicles, men circumvent reliance on women for validation while gaining stature in the eyes of other men or at least other car guys.

## Conclusion

The body of memes presented in this paper reveals that masculinity can be expressed in material resources wherein a car embodies characteristics of hegemonic masculinity, namely features that car guys seek to emphasize. Cars also may symbolically substitute for women, projecting masculine control and independence, an antidote to women as the proverbial “ball and chain” that is a metaphor for women who constrain men's freedom and control the fulfillment of their libidinous needs. In that sense, showy cars can celebrate single life, nostalgia for single life, and the ability to spend uninterrupted time on pursuits that affirm masculinity. While this desideratum is not novel, its sustained resonance as expressed in memes shows resistance to gender parity and the long road still ahead.

Rather than expend energy and risk rejection by dating, a young man can gain status from cars. This perspective is visible in memes, especially those that belittle women to justify why cars are an attractive substitute for women. These memes ultimately reflect ambivalence about committed relationships and the attendant responsibilities that detract from leisure time, freedom, and independence. By defining their masculinity relative to their cars, car guys ultimately feel a greater sense of control compared to depending on women to validate virility. In the future, car guy memes may become less focused on themes of escaping responsibility and asserting masculinity. Similarly, the genre of “car guys” could evolve to be more gender inclusive, to reflect all individuals who are car aficionados, removing aspects of gender from the insider–outsider appeal of the subculture.

## Data availability statement

The original contributions presented in the study are included in the article/supplementary material. Further inquiries can be directed to the corresponding author.

## Author contributions

The author confirms being the sole contributor of this work and has approved it for publication.

## Footnotes

^1^Some car guys believe the Camaro, Barracuda, Challenger, and Firebird are pony cars, not muscle cars, based on the first Ford Mustang (1964), a classic pony car (Drummond, 2021).

^2^Various car guy memes emasculate Honda drivers, perhaps because this Japanese brand contrasts with American-made muscle cars. E.g., (1) Chuck Norris can do anything... but make people respect a Honda. 2) [on Condescending [Willy] Wonka template]: VTEC kicked in? Tell me all about your fast and furious lifestyle [VTEC is Honda's technology to make its cars faster]. (3) What size engine is in your Honda? 1.8 liter? My Mountain Dew has 2 l.

^3^Stock racing cars cannot be legally driven on streets because they lack required safety features. However, they are more relatable than exorbitant race cars; in 2021, F1 teams acceded to a cap of $145 million (contrasting with $20 million for the IndyCar series cap) (Patel, 2021).

^4^The car used in the film is a trophy of sorts, that sold for $3.74 million in 2020. In fact, McQueen, had unsuccessfully tried to buy the car (in 1977, a few years prior to his death) (Garrett, 2020), revealing its symbolic importance.

^5^“Odd Rod” trading cards of the 1970s feature various hot rods and muscle cars with menacing drivers (like Speed Demon, Ram Charger, King Cougar and Big Daddy). One of the few women drivers (on card #31) is Drag-on-Lady, a play on the term dragon lady, a domineering woman. She is portrayed with a phallic, witchlike nose and chin, gripping a ball connected to a stick shift, that is, a castrating old witch appropriating hegemonic power.

^6^Expressions applied to humans “to run out of gas,” “blow a gasket” or “get a tune up” (e.g., by going to a doctor) (Morgan, 2009) provide linguistic support for perceptions of cars as pseudo-living beings. The term “muscle car,” also connotes a human body, a term first used in 1966 to refer to “an American-made two-door sports car with a powerful engine” (Muscle Car, 2021).

^7^Car Lashes to decorate a car's headlights with fake lashes make the car's “eyes” more prominent, a product targeting women: “Since exploding online in 2010, CarLashes^®^ have gone from a girly trend to a diva necessity” (https://carlashes.com/).

^8^The term boot refers to a trunk in the UK, reflecting polysemic meaning. However if the front end of a car is a head, then the rear end of the car may not always be a rear-end equivalent, but rather symbolize a foot (as in the expression, “from head to toe”).

^9^President John F. Kennedy “had a ‘thing’ for cars. Like most red-blooded American males, he appreciated big, *shiny*, powerful automobiles,” reflected in his Corinthian white 1961 Ford Thunderbird convertible (Darby, 2013 [emphasis added]). Shininess, like knights in shining armor, is achieved by car waxing that links car's phallic power to sexual readiness (Dundes et al., 2018), a common goal of car “buffs”.

^10^In Australian band Midnight Oil's 1987 song “Bullroarer,” a children's version of a bullroarer was used (a ruler tied to a piece of rope). The band respected the bullroarer as a “sacred instrument” making sounds “only initiated men are supposed to hear” (Lawrence, 2016, pp. 171–172).

^11^This theme is not new, reflected in Queen's song “I'm in Love with my Car” (1975) with lyrics describing how a man told his girl he'd “have to forget her” given his preference for his car that does not “talk back”.

^12^The number of other expressions that treat women as sex objects speaks to the pervasiveness of this mindset: “smash and dash,” “F$%^*^ and chuck,” “copulate and depopulate,” “tap it and gap it,” “screw and scram,” “pound and bound,” “hump/pump and dump,” “toot it and boot it,” “conceive and leave,” “bust a load and hit the road.” The last example is particularly relevant to cars' association with freedom from commitment.

^13^The appeal of a racecar (a palindrome) is apparently so palpable that it arguably serves as a form of “autoeroticism” (pun intended).

^14^The car's tremendous engine power is linked to its initials, LT meaning longtail, a likely nod to tail as sexually suggestive, including one meaning as slang for sexual intercourse.
